# An innovative tool to prioritize the assessment of investigational COVID-19 therapeutics: A pilot project

**DOI:** 10.14745/ccdr.v50i10a04

**Published:** 2024-10-03

**Authors:** Lizanne Béïque, Savannah Clarke, Mina Azad, Elaha Sarwar, Margaret Gale-Rowe, Stacy Sabourin, Cheryl Marinsky, Jacqueline Arthur

**Affiliations:** 1 Infectious Diseases and Vaccination Programs Branch, Public Health Agency of Canada, Ottawa, ON

**Keywords:** decision support techniques, therapeutic evaluation, investigational therapeutics, COVID-19, prioritization

## Abstract

**Background:**

As the COVID-19 pandemic unfolded, hundreds of investigational COVID-19 therapeutics emerged. Maintaining situational awareness of this extensive and rapidly evolving therapeutic landscape represented an unprecedented challenge for the Public Health Agency of Canada, as it worked to promote and protect the health of Canadians. A tool to triage and prioritize the assessment of these therapeutics was needed.

**Methods:**

The objective was to develop and conduct an initial validation of a tool to identify investigational COVID-19 therapeutics for further review based on an efficient preliminary assessment, using a systematic and reliable process that would be practical to validate, implement and update. Phase 1 of this pilot project consisted of a literature search to identify existing COVID-19 therapeutic assessment prioritization tools, development of the Rapid Scoring Tool (RST) and initial validation of the tool.

**Results:**

No tools designed to rank investigational COVID-19 therapeutics for the purpose of prioritizing their assessment were identified. However, a few publications provided criteria to consider and therapeutic ranking methods, which helped shape the development of the RST. The RST included eight criteria and several descriptors (“characteristics”). A universal characteristic scoring scale from −10 to 10 was developed. The sum of all the characteristic scores yielded an overall benefit score for each therapeutic. The RST appropriately ranked therapeutics using a systematic, reliable and practical approach.

**Conclusion:**

Phase 1 was successfully completed. The RST presents several distinct aspects compared with other tools, including its scoring scale and method, and capacity to factor in incomplete or pending information. It is anticipated that the framework used for the RST will lend itself to use in other dynamic situations involving many interventions.

## Introduction

### Background

At the beginning of the pandemic, the rapid global transmission of SARS-CoV-2, the virus that causes COVID-19, prompted extensive research into a range of treatment options. As the pandemic unfolded, hundreds of investigational (i.e., prior to market authorization) pharmaceutical COVID-19 therapeutics emerged ((1)). Maintaining situational awareness of this extensive and rapidly evolving therapeutic landscape represented an unprecedented challenge for the Public Health Agency of Canada (PHAC), as it worked to promote and protect the health of Canadians ((2)). A timely and thorough assessment of all investigational therapeutics was not feasible. Therefore, a practical tool to systematically, reliably and efficiently triage and prioritize the assessment of these therapeutics was needed to help inform their potential applicability for Canada.

To identify existing COVID-19 investigational therapeutic assessment prioritization tools, a literature search was conducted in Ovid MEDLINE® with the assistance of a PHAC librarian, using the focused search concepts “decision support techniques,” “COVID-19 therapeutic treatment or assessment” and variations of their terms. A total of 302 articles were identified; 46 were deemed relevant and these were reviewed. The search identified no tools designed to rank investigational COVID-19 therapeutics to prioritize their assessments. However, several publications provided criteria to consider when conducting health technology assessments or making therapeutic formulary decisions ((3–9)). Furthermore, some of these publications and their references featured different therapeutic ranking methods and evaluation frameworks ((4,7,9–16)). Although they had important limitations (e.g., required a pre-defined list of therapeutics with known properties, complex to implement or adapt quickly), certain elements, such as their assessment criteria and use of positive and negative scoring, were found relevant to incorporate into a tool that PHAC developed in the fall of 2022. This article reports on the first phase of this pilot project to develop what has become known as the Rapid Scoring Tool (RST).

### Objective

To develop and conduct an initial validation of a tool to identify investigational COVID-19 therapeutics for further assessment, based on an efficient preliminary review, using a systematic and reliable process that would be practical to validate, implement and update.

### Intervention

#### Setting

During the pandemic, a team of four individuals from the PHAC COVID-19 Therapeutics team was formed to develop the RST. The members had backgrounds in critical appraisal, clinical and research pharmacy, therapeutic evaluation, program evaluation, epidemiology, immunology and public health. Investigational COVID-19 therapeutics were identified primarily from a daily scan of key COVID-19 sources of information (e.g., updates and pre-prints of key COVID-19 trials) and ClinicalTrials.gov. The RST was developed using Microsoft Excel®.

#### Intervention

The pilot project had two phases:

· Phase 1: Development (stages one and two) and initial validation (stage three) of the RST

· Phase 2: Further validation and enhancement of the RST

**Stage one: Design the RST.** The RST team developed the RST, which included defining the decision problem it was intended to address ((13–15)), the broad categories or “criteria” that would be used to assess therapeutics (e.g., safety), and more precise descriptors or “characteristics” within each criterion. The criteria and characteristics were developed based on literature findings, feasibility of implementation and over a dozen internal discussions with stakeholders, both within and outside of the COVID-19 Therapeutics team, involved in the assessment and monitoring of therapeutics (i.e., medical advisors, managers, epidemiologists, policy analysts and research analysts). Next, a “characteristic” scoring scale was constructed based on the decision problem. This universal scale was used to assign a score to each characteristic. For each therapeutic, an overall perceived benefit (“overall benefit”) score was calculated by summing the scores of all the characteristics that applied to that therapeutic.

**Stage two: Pilot test the RST.** During stage two, therapeutics were entered into the RST and ranked by their “overall benefit” score to identify those to assess more thoroughly. Two members of the RST team independently selected the appropriate characteristics (one for each criterion) from the list of possible characteristics, using key sources of information. All discrepancies were resolved through discussion with a third member until full agreement among the three members was reached. When adjustments to the criteria, characteristics and/or their associated scores were required, an iterative consensus approach within the RST team was used, with input from stakeholders, to validate and maintain internal consistency (i.e., alignment and coherence among the RST components). Face validity of the ranking, internal consistency and reliability of the RST were deemed to have been achieved once 10 consecutive therapeutics had been entered without discrepancies (i.e., the need to involve a third member of the team) or the need to adjust the RST and the ranking was deemed appropriate by the members of the RST team.

**Stage three: Conduct an initial validation of the RST.** This stage consisted of further validation of the RST using the input from three members of the COVID-19 Therapeutics Team who had not used the RST to assess individual therapeutics. Together, they had critical appraisal skills, medical, nursing and public health backgrounds. They were provided with detailed information on 15 randomly selected therapeutics in the RST (using the RAND function of Microsoft Excel) They were given time to ask questions and deliberate, and asked to indicate their level of agreement or disagreement (using a Likert scale) with the RST’s ordinal ranking of these therapeutics (i.e., which therapeutic ranked first, second, etc.). They were also asked to provide statements describing the intervals between rankings (e.g., therapeutic A is clearly of greater overall benefit compared with therapeutic B; therapeutics C and D offer very similar overall benefit). The rankings were considered validated (‘’appropriate’’) if at least two of the three individuals agreed or strongly agreed (consensus agreement) with the ordinal ranking of therapeutics and on 75% or more of the 12 ranking statements. This consensus agreement approach was adopted to leverage the benefits of collaborative decision-making, while mitigating risks associated with individual biases; the 75% threshold was considered practical and meaningful to describe substantial consensus.

#### Outcome measures

**Table 1** provides the list of outcome measures and stages during which they were assessed.

**Table 1:** Outcome measures, description and stage

Objective	Outcome measure	Description	Stage(s)
Development of the RST	Systematic nature of the RST	The RST’s systematic nature was assessed based on: the structure (logical and intuitive sequence and configuration), operationality (clarity of definitions), non-redundancy (no duplicates) and mutual independence (without overlap) of the criteria; characteristics and characteristic scores of the RST ((15)); and its internal consistency.	1 and 2
Development of the RST	Practicality of the RST	The practicality of the RST was assessed based on the feasibility of implementation (whether the RST could be set up using Microsoft Excel), use (ease with which members can select and enter information into the RST) and adaptation (ease with which the criteria, the characteristics and their scores could be modified in accordance with the changing pandemic environment).	1 and 2
Development of the RST	Intra-rater and inter-rater reliability	The intra-rater reliability (consistency in the selection of the characteristics for a same therapeutic by a same RST team member over time, for example, when updating information for a therapeutic) and inter-rater reliability (consistency in the selection of the characteristics for a same therapeutic between members of the RST team for every therapeutic entered in the RST).	2
Development of the RST	The time required to conduct a preliminary assessment of each therapeutic	The time was assessed once the RST team had become accustomed to the RST (after having entered approximately 15 therapeutics in the RST). The aim was for the RST to enable the preliminary assessment of each therapeutic within 30 minutes.	2
Development and initial validation of the RST	Appropriateness of ranking of therapeutics	The appropriateness of ranking of therapeutics was assessed based on face validity of the ranking of therapeutics.	2 and 3

Abbreviation: RST, Rapid Scoring Tool

### Outcomes

#### Design of the Rapid Scoring Tool

The decision problem pertained to the need to efficiently triage and prioritize the large number of investigational COVID-19 therapeutics for further assessment, based on a preliminary assessment of their perceived benefit, within the Canadian context. The criteria included in the RST at the time of writing, and the elements that were used to develop the characteristics for each criterion, are listed in **Table 2**.

**Table 2:** Criteria and elements considered to develop their characteristics

Criteria	Elements considered to develop the characteristics
Quality of evidence	Phase of the study, study design, availability of results and whether they were peer-reviewed and important limitations (e.g., limited generalizability of the results)
Clinical impact	Type of outcomes, the classification of outcomes as either primary or secondary, magnitude of the impact and its statistical significance
Safety data	Adverse events, warnings and precautions, contraindications and drug interactions
Patient preference	Benefits and harms of the therapeutic, route of administration, ease of access to the therapeutic (for outpatient therapeutics) and frequency of dosing
Availability of authorized treatment alternatives for the same broad target patient population	Number of authorized treatment alternatives. Broad target patient populations: outpatients, inpatients not in an intensive care unit, inpatients in an intensive care unit, patients with post COVID-19 condition
Authorization status in Canada	Presence or absence of an authorized indication other than the one being studied
Regulatory status in other jurisdictions	Regulatory status in the United States, Europe, Australia and other select countries with stringent regulatory authorities
Domestic therapeutic development landscape	Current or past Canadian funding, study sites in Canada and geographical location of the manufacturer

**Figure 1:**
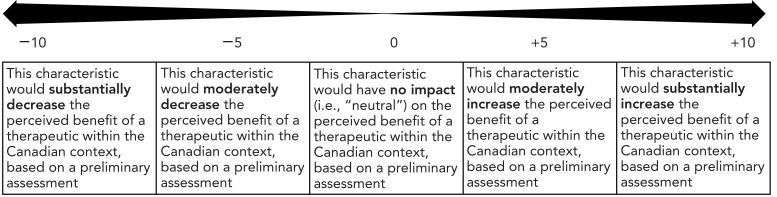
Scoring scale of the characteristics

**Figure 1** shows the scale developed and used to assign a score to each characteristic, with scores ranging from −10 to +10. In most cases, characteristics had only a moderate effect on the perceived benefit of a therapeutic and, as a result, most scores were in the −5 to +5 range.

**Table 3** provides an example of a criterion, its associated characteristics and their scores from the RST. For example, if a therapeutic was shown to be associated with serious liver toxicity during a Phase 3 trial leading to a serious warning and precaution, characteristic 4, “Serious warnings and precautions (…)” would be the characteristic selected for the safety criterion for that therapeutic. During the development of the tool, it was decided that this characteristic would decrease the perceived benefit of a therapeutic having this characteristic, within the Canadian context and based on a preliminary assessment, and be assigned a score of −2 (as per Figure 1).

**Table 3:** Example of the safety criterion, its characteristics and their scores

Safety criterion characteristics	Characteristic's score
**Characteristic 1:** None of potential significance from a Phase 3 trial or real-world evidence (i.e., no AEs or mild to moderate AEs; no significant type or number of DIs, warnings and contraindications)	2
**Characteristic 2:** Unknown, but probably no AEs of significance (i.e., no AEs or mild to moderate AEs; no significant type or number of DIs, warnings and contraindications)	1
**Characteristic 3:** Unknown	0
**Characteristic 4:** Serious warnings and precautions or indication restricted because of significant safety concerns (e.g., therapeutic authorized for COVID-19 in another jurisdiction, for a non-COVID-19 indication in Canada or for a COVID-19 indication if being assessed for post-COVID-19 condition)	−2
**Characteristic 5:** Unknown, but probably some of significance (i.e., at least one of: significant AEs, DIs, warnings or contraindications or a serious AE of particular concern)	−3

Abbreviations: AE, adverse event; DI; drug interaction

## Methods

### Implementation of the Rapid Scoring Tool

**Figure 2:**
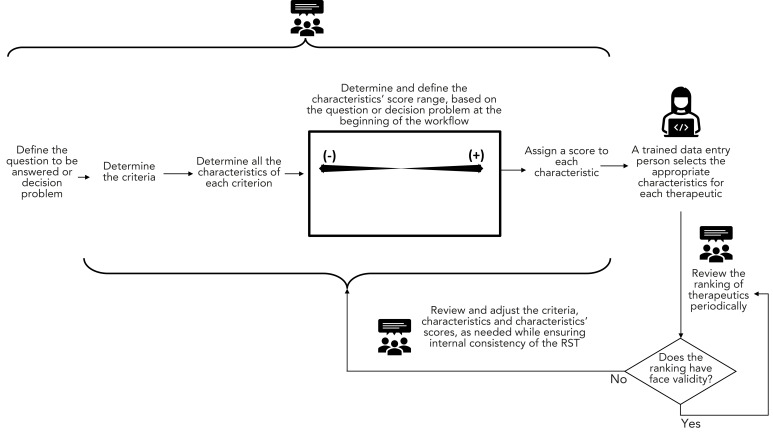
Simplified overall workflow of the Rapid Scoring Tool Abbreviation: RST, Rapid Scoring Tool

**Figure 2** depicts a simplified version of the workflow used for developing the RST during Phase 1. Some therapeutics could be excluded from further assessment based on a single characteristic. These characteristics of exclusion were assigned a score of −100 to ensure that therapeutics with these characteristics have low “overall benefit” scores and would not be among the top-ranked therapeutics for further assessment. Characteristics of exclusion are shown in **Box 1**. Given the rapidly changing pandemic environment, all therapeutics were reassessed periodically (whenever new information arose from daily scans of key COVID-19 resources or every six months, whichever occurred first).

Box 1 **Characteristics of exclusion**

Abbreviation: PHAC, Public Health Agency of Canada

## Results

### Outcome measures of the Rapid Scoring Tool

After approximately 30 therapeutics were entered in the RST during stage two, the outcome measures, including appropriateness of ranking and the systematic nature, reliability and practicality of the RST, as well as the time required for completing a preliminary assessment, had been met. A standard operating procedure was developed to ensure ongoing consistency using the RST. Appropriateness of ranking was also met during stage three. Consensus agreement was reached for the ordinal ranking of all therapeutics, and for 10 of the 12 (83%) statements describing the intervals between rankings; disagreements pertained to two therapeutics. Adjustments were made to the RST, and the overall ranking of these therapeutics relative to the others was reviewed until consensus was reached. Ten months into Phase 1, 69 investigational COVID-19 therapeutics had undergone a preliminary assessment using the RST.

## Discussion

In a dynamic pandemic environment, it was challenging to identify therapeutics (with incomplete information) in a timely manner for further assessment to enhance situational awareness. The RST enabled this through a continuous iterative process to update and validate the criteria, characteristics and characteristic scores, as well as its unique scoring scale. The RST scoring scale standardized all characteristic scores and directly incorporated the concept of “importance” that other tools typically address by assigning weights to criteria ((7,13–15)). **Appendix** was developed to provide further details on these key aspects of the RST, as well as some of their benefits compared with other commonly-used tools, such as the System of Objectified Judgment Analysis based tools ((7,17–19)) and other Multi-Criteria Decision Analysis-based tools ((11,16,20)).

In addition to its primary role, identifying therapeutics for further assessment, the RST served as a structured repository for key information pertaining to the therapeutics, which facilitated timely updating with new information and monitoring. This further enhanced situational awareness of the investigational therapeutic landscape.

### Limitations

The RST has limitations that are inherent to the context in which it was developed and operationalized. How they were considered and mitigated is described below. To optimize the efficiency of the preliminary assessment, the RST relied on a subset of assessment criteria used in more thorough reviews. For example, implementation factors were not part of the RST, as this information was often not available or could not be determined rapidly. A different subset of assessment criteria might have affected the ranking of therapeutics. The initial validation during stage three, however, suggested that the subset of criteria and characteristics selected was adequate for identifying therapeutics for further assessment.

The score assigned to each characteristic was agreed upon by a specific group of individuals. A different group might have assigned different scores, which could have affected the ranking of therapeutics. This limitation is inherent to any decision-making process ((18,21)) and was mitigated by involving individuals with different backgrounds and roles in the design and validation of the RST.

The initial validation of the RST was led by the RST team, which might have affected the results. Several steps were taken to mitigate this potential limitation, such as using a structured presentation with questions that were carefully worded for clarity and neutrality, and efforts to avoid motivational biases.

#### Implications and next steps

The RST enabled timely identification of therapeutics to be assessed more thoroughly, as well as efficient tracking of the therapeutic landscape in an evolving environment. Its iterative approach ensured that it integrated the most up-to-date information on the criteria, characteristics, scores, and therapeutics. By nature of this design, stages two and three of Phase 1 will be repeated periodically.

Phase 2 of this pilot project will consist of assessing the validation and reliability of the RST with additional therapeutics and stakeholders, and formal statistical and sensitivity analyses. It is anticipated that an adapted framework would lend itself to other dynamic situations involving many interventions.

## Conclusion

Phase 1 of the pilot project was successful. The RST enabled a systematic, reliable and efficient prioritization of investigational COVID-19 therapeutics for further assessment and enhanced situational awareness of the emerging therapeutic landscape during a dynamic pandemic. The RST presents several distinct aspects compared with other tools, including its scoring scale and method, and capacity to factor in incomplete or pending information.

## Appendix

**Table A1:** Key aspects and benefits of the Rapid Scoring Tool compared with commonly used tools^a^

Rapid Scoring Tool	Commonly used scoring tools
The scoring scale includes negative values, zero, and positive values. Negative values are assigned to characteristics that are undesirable (e.g., serious adverse events), and positive values to characteristics that are desirable (e.g., robust clinical trial design). A characteristic that is neither desirable nor undesirable is assigned a value of zero as it would neither increase nor decrease the perceived benefit a therapeutic with that characteristic would have (Figure 1). It is more intuitive to assign negative scores, rather than low positive scores, to undesirable characteristics.	The scoring scales typically start at zero and only include positive values, regardless of whether the characteristic is desirable or undesirable.
The interpretation of a characteristic score remains consistent, regardless of the characteristics involved. The scores of the characteristics are standardized as they always represent the same measure. The scores reflect the impact a characteristic would have on the overall perceived benefit a therapeutic with that characteristic would have (Figure 1). This aspect helps ensure internal consistency of the scores among different characteristics. All the characteristic scores were assigned based on the answer to this question: “How would this characteristic impact the perceived benefit of this therapeutic?” (Figure 1). If a new characteristic is added and assigned a score, one could ensure internal consistency by asking: “Would a therapeutic with this new characteristic have the same perceived benefit as another therapeutic with a different characteristic with the same score?”	The interpretation of a score often varies, depending on what is being assessed. Although these scores are sometimes then converted using a common scale, it is more challenging to ensure internal consistency of the tool. For example, a score of 5 for a safety characteristic may not have the same meaning as a score of 5 for a dosage characteristic, or an undesirable characteristic of a criterion could have the same score as a desirable characteristic from a different criterion.
The RST can include characteristics of exclusion that are assigned a negative score that cannot be balanced out by the scores of other, desirable, characteristics. As a result, a therapeutic with a characteristic of exclusion would get ranked at the bottom of the list of therapeutics. For example, if a therapeutic had no activity against a dominant circulating COVID-19 variant, the RST would rank it very low on the list of therapeutics, regardless of how high its scores are for other characteristics. Therapeutics were periodically reassessed to ensure their selected characteristics reflected the most current information.	Other tools typically do not include characteristics of exclusion. Therapeutics with a very undesirable characteristic could still be ranked among the therapeutics at the top of the list of therapeutics of interest if other, desirable, characteristics override the score of that very undesirable characteristic.
No weights are assigned to criteria. This ensures that the impact of characteristics of exclusion and “outstanding” characteristics have the intended impact on the overall perceived benefit of a therapeutic, based on a preliminary assessment.	Other tools typically assign weights to criteria to indicate their importance relative to that of the other criteria. Criteria are umbrella terms that include several possible characteristics. Assigning weights to criteria can be problematic, especially in an environment where new therapeutics with new characteristics emerge, because the “importance” of a criterion is dependent on its characteristics. A scoring tool that assigns weights to criteria would not fare well in handling therapeutics with a characteristic of exclusion or an “outstanding” characteristic. This is because the impact of these characteristics on the perceived “overall benefit” of the therapeutic would be fixed and pre-determined by the weight of their criterion. For illustrative purposes, we will use a simplified scoring tool with only four criteria: quality of evidence, clinical impact, safety and dosage. Quality of evidence is assigned a weight of 40 points, clinical impact 30 points, safety 20 points and dosage 10 points, for a total of 100 points. The weight of the dosage criterion was determined according to whether the dosage of a therapeutic is, for example, once daily for 10 days, twice daily for five days, or three times daily for three days. This criterion was determined to be of low importance relative to the other criteria and was given a weight of 10% in the assessment. A new dosage, for example once every month, then becomes available and is deemed to be of particular benefit. The impact of this characteristic will be limited by the weight of its criterion (i.e., it will only be able to account for a maximum of 10 out of 100 points). As a result, this new characteristic may not be well-reflected in the overall perceived benefit of this therapeutic.
The RST can incorporate incomplete or unknown information, because of the design of the scoring scale (Figure 1). For example, the RST has a characteristic for therapeutics with an “unknown clinical impact” that was assigned a score of zero because that characteristic had no impact on the perceived benefit of this therapeutic. When results became available, the characteristic (and its associated score) was updated.	Other scoring tools are typically only able to consider a set of therapeutics with complete information on each therapeutic.
The scores of the characteristics could be easily adjusted as the pandemic environment evolved or new information became available, and their impact on the overall ranking relative to other therapeutics, quickly seen.	These tools typically assess therapeutics at a single point in time and updating them based on new information can be cumbersome and a lengthy process.

Abbreviation: RST, Rapid Screening Tool

^a^ For example, the System of Objectified Judgment Analysis based tools (7,17–19) and other Multi-Criteria Decision Analysis-based tools (11,16,20)
